# The Role of OCT in Follow-Up of Fungal Keratitis Caused by *Beauveria bassiana* in Contact Lens Wearer

**DOI:** 10.3390/diagnostics14131382

**Published:** 2024-06-28

**Authors:** Cristina Martínez-Gil, María José Roig-Revert, Ester Fernández-López, Rosa María González-Pellicer, Juan José Camarena-Miñana, Cristina Peris-Martínez

**Affiliations:** 1 Fundación de Oftalmología Médica de la Comunitat Valenciana, 46015 Valencia, Spain; 2 Department of Microbiology, Hospital Univeristari Doctor Peset, 46017 Valencia, Spain; 3 Department of Microbiology and Ecology, Universitat de Valencia (UV), 46010 Valencia, Spain; 4 Department of Surgery-Ophthalmology, Universitat de Valencia (UV), 46010 Valencia, Spain

**Keywords:** keratitis, fungal, optical coherence tomography

## Abstract

A 25-year-old Caucasic female was referred to our clinic after suffering from infectious keratitis in the right eye for a month. The patient was a contact lens user and had no history of ocular trauma. Furthermore, the patient did not report any relevant antecedent. The main complaint was intense photophobia and pain. Infectious keratitis remains one of the main complications of contact lens wear and can become a therapeutic challenge in some patients. Although the most frequent causal agent is bacterial, other causes such as herpes virus, *Acanthamoeba* or fungi should be considered when antimicrobial therapy does not work as expected clinically. Fungal keratitis normally appears on previously damaged corneas, but it can also develop in contact lens wearers. *Beauveria bassiana* is an unusual pathogen which has been diagnosed more frequently lately per the clinical reports in the last 30 years, so it can be included in the diagnostic scheme when a fungal keratitis is suspected. In clinical management, AS-OCT may be a functional tool to assess the evolution and monitor the response to microbial agents and surgery. Although more studies are needed, some characteristic features have been described and can help to diagnose a fungal keratitis against other infections. AS-OCT can also play an important role in monitoring the corneal scarring after the keratitis episode, and it may be useful to plan post-infection therapy for visual rehabilitation.

**Figure 1:**
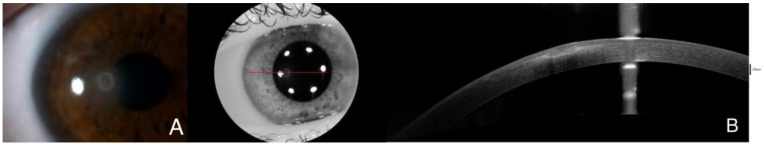
Slit-lamp examination demonstrates a 3 × 3 mm paracentral ulcer affecting epithelium and anterior stroma with 2 mm perilesional infiltrate. No signs of inflammation were identified on the anterior chamber (**A**). Initially, AS-OCT displayed Bowman layer disruption with stromal and epithelial edema, along with stromal hyperreflectivity, which may indicate an inflammatory process taking place (**B**). A corneal scrapping sample for microbiologic cultures was obtained and the patient started receiving hourly fortified topical treatment with Vancomycin and ceftazidime, along with topical voriconazole every two hours and ciprofloxacin ointment once a day. The best corrected visual acuity in that moment was 0.6 in the right eye. The red line is correspondene of the en face picture with the selected section on the AS-OCT.

**Figure 2:**
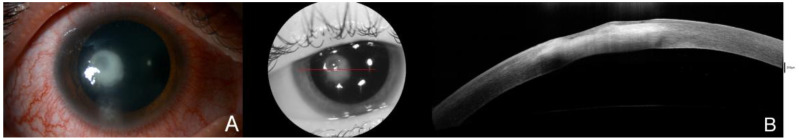
(**A**). Clinical evolution with endothelial infiltrates and expansion of the infiltrate, as well as corneal necrosis hyperreflective in AS-OCT, with small cystic spaces next to the epithelium. The initial culture from the corneal sample returned positive for *Streptococcus oralis* resistant to eritromicine, clindamicine and tetracycline. Due to these results and a torpid clinical course without symptomatic improvement, appearance of endothelial deposits and impairment of stromal edema in AS-OCT with hyperreflectivity reaching deeper layers (**B**) and BCVA loss to 0.3, the treatment was updated with interruption of the fortified topical antibiotics, which were switched to moxifloxacin and linezolid. Natamicyn was added to the treatment in substitution of voriconazole. Two days later, the endothelial deposits disappeared and the patient related symptomatic improvement, but a week after, the BCVA in the right eye was severely reduced to 0.05. The red line is correspondene of the en face picture with the selected section on the AS-OCT.

**Figure 3:**
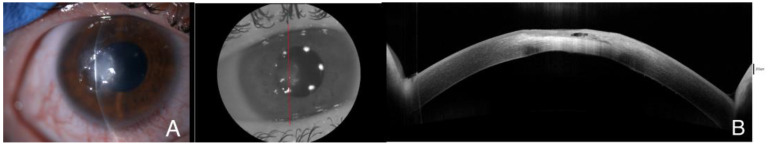
Corneal debridement and biopsy were performed, along with intrastromal infiltration with amphotericine and cefuroxime. A total of 24 h after the procedure, the infiltrate appeared smaller with a large epithelial defect in both clinical examination (**A**) and AS-OCT (**B**), which also displayed intrastromal cysts and hyperreflectivity in both stroma and epithelium, as well as edema and cystic spaces, along with tissue defects due to the surgical procedure. The red line is correspondene of the en face picture with the selected section on the AS-OCT.

**Figure 4:**
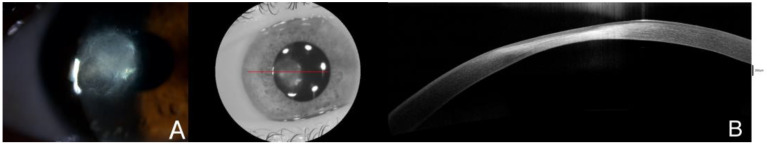
Four days after surgery, the infiltrate kept becoming smaller, and as soon as the epithelial defect disappeared, corticosteroid therapy was initiated. After clinical improvement, slip lamp examination (**A**) demonstrated a reduced infiltrate with epithelial healing in AS-OCT (**B**), although hyperreflectivity remained due to the beginning of the leukomatous phase. Due to corneal scarring, stromal thinning could also be found in AS-OCT. The red line is correspondene of the en face picture with the selected section on the AS-OCT.

**Figure 5:**
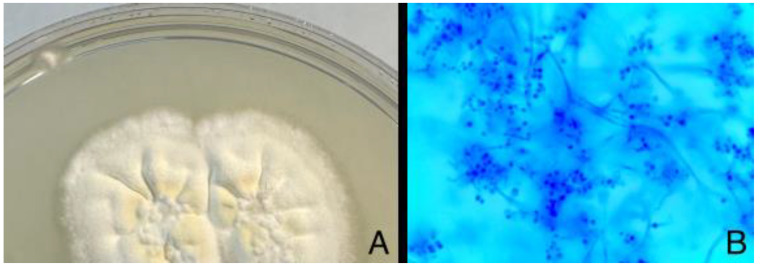
Concurrently, after ten days, the corneal scraping culture on Sabouraud dextrose agar was found positive for white cottony mold colonies. *Beauveria bassiana* was identified by MALDI-TOF (Bruker) and macroscopic–microscopic features of the mold. The antifungal susceptibility of this filamentous fungus was evaluated by Etest (BioMèrieux). The strain was resistant to fluconazole (MIC > 256 µg/mL) and sensitive to voriconazole (MIC 0.125 µg/mL). Furthermore, RT-PCR of the sample also detected Herpes zoster (VZV) DNA. Subfigure (**A**) is the mascroscopic appearance of the moldy colony and (**B**) is the microscopic appearance. Topical therapy was then updated to linezolid, amphotericine and chloramphenicol, along with oral acyclovir. This therapeutic scheme was kept for a month and slowly tapered with significant clinical improvement. Fungal keratitis is a sight-threatening infection. Several risk factors have been identified, such as vegetal trauma, contact lens use, chronic treatment with topical steroids, corneal transplantation or immunodeficiency conditions. Fungal agents can also appear on exposure keratitis or herpes simplex virus keratitis. *Beauveria bassiana* has been described as an opportunistic filamentous fungus which causes keratitis in patients with antecedents of eye surgery or trauma, although it has been described as an infectious agent in contact lens use alone—mostly therapeutic, or in bullous keratopathy. When it appears as a causal agent in eye disease, it usually does so as a superficial infiltrate, although severe infections such as necrotizing sclerokeratitis and endophthalmitis have been described [1,2]. The patient in this case report was diagnosed with fungal keratitis weeks after the symptoms began. The lack of acknowledged ocular pathology led us to think the predisposing cause of the infection was the use of contact lenses alone, although herpes zoster viridae was positive later in the culture—probably being a contributing factor to developing this corneal pathology. *Beauveria bassiana* infections can require more than one antifungal topical agent—usually voriconazole and natamycin, as well as systemic antifungal therapy. Surgical debridement has been also used to reduce microbial load and help the topical therapy penetrate deeper. It appears to be a rising agent of infection, since it has been reported in less than 30 cases from 1984, mostly in the last decade [1]. The case we present responded to antifungal treatment and surgical debridement. Still, it had a torpid clinical course with initial improvement with antibiotic therapy and posterior functional and symptomatic deterioration, which had already been described previously in the literature [3]. AS-OCT was a helpful tool to assess the clinical evolution and to correlate the clinical signs to images, with important changes in stromal reflectivity during the episode and epithelial changes demonstrating healing by the end of the episode.

**Figure 6:**
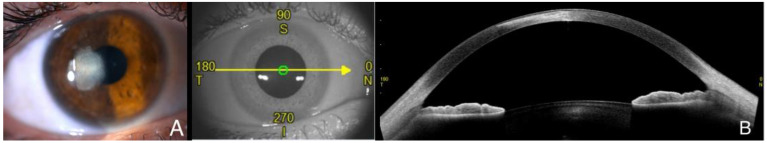
Clinical examination of the eye (**A**) and AS-OCT correlation (**B**) months after the episode. Antimicrobial treatment was tapered until complete resolution of the symptoms. At the end of the episode, the clinical examination demonstrated a paracentral leukoma, and AS-OCT displayed regular hyperreflectivity in that area in a thinned stroma with complete restoration of the epithelium. The clinical course was monitored with physical examination in slit lamp and imaging using AS-OCT scans. In those images, the main findings were increased corneal thickness, hyperreflective stromal lesions and appearance of cystic spaces. These findings have been described in fungal keratitis monitored by AS-OCT in previous reports [4] and can be a useful tool to consider stromal affectation. Although it is not a routine exam in most cases, when compared to other tests such as in vivo confocal microscopy, AS-OCT can be helpful to assess the severity of the corneal infection by measuring the extension and depth of the infiltrate and identifying other features, such as corneal haze and epithelial defect. Specifically, in infectious keratitis with corneal opacity, it can be used as a tool to diagnose endothelial plaques and register the response to antimicrobial treatment [5]. It can also be useful to perform a differential diagnosis from other microbial causes with characteristic features in AS-OCT—for instance, *Acanthamoeba* spp. keratitis. Once the infectious episode has ended, corneal scarring can be evaluated with AS-OCT by performing a corneal densitometry and topography. The arrow is correspondence of the en face picture with the selected section on the AS-OCT, the letters are N nasal, S superior, T temporal and I inferior, and the numbers are the gradation of the 360 degrees of the cornea.

**Figure 7:**
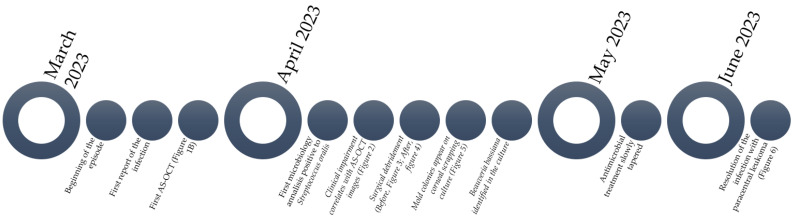
Flowchart summarizing the whole episode and its timeline and image documentation.

## Data Availability

No new data were created or analyzed in this study. Data sharing is not applicable to this article.
